# Performance of the Bethesda System for Reporting Thyroid Cytology in Multi-Institutional Large Cohort of Pediatric Thyroid Nodules: A Detailed Analysis

**DOI:** 10.3390/diagnostics12010179

**Published:** 2022-01-12

**Authors:** Sule Canberk, Helena Barroca, Inês Girão, Ozlem Aydın, Aysun Uguz, Kıvılcım Erdogan, Ebru Tastekin, Massimo Bongiovanni, Paula Soares, Valdemar Máximo, Fernando Schmitt

**Affiliations:** 1 Instituto de Investigação e Inovação em Saúde (i3S), University of Porto, Rua Alfredo Allen 208, 4200-135 Porto, Portugal; psoares@ipatimup.pt (P.S.); vmaximo@ipatimup.pt (V.M.); 2 Institute of Molecular Pathology and Immunology of the University of Porto (Ipatimup), Rua Júlio Amaral de Carvalho 45, 4200-135 Porto, Portugal; fschmitt@ipatimup.pt; 3 Abel Salazar Biomedical Sciences Institute (ICBAS), University of Porto, Rua Jorge de Viterbo Ferreira 228, 4050-313 Porto, Portugal; 4 Department of Pathology and Oncology, Centro Hospitalar São João, Alameda Professor Hernâni Monteiro, 4200-319 Porto, Portugal; hbarroca@gmail.com; 5 Department of Biomedicine and Medicine, University of Algarve, 8005-139 Faro, Portugal; ines_girao@hotmail.com; 6 Department of Pathology, Medical Faculty, Acibadem University, Istanbul 34684, Turkey; ozlemaydin66@hotmail.com; 7 Department of Pathology, Medical Faculty, Çukurova University, Adana 01330, Turkey; druguz@gmail.com (A.U.); kerdogan@cu.edu.tr (K.E.); 8 Department of Pathology, Medical Faculty, Trakya University, Edirne 22030, Turkey; ebrutastekins@gmail.com; 9 Synlab Pathology, Rue du Liseron 5, 1006 Lausanne, Switzerland; massimo.bongiovanni@synlab.com; 10 Department of Pathology, Faculty of Medicine of the University of Porto (FMUP), Alameda Professor Hernâni Monteiro, 4200-319 Porto, Portugal; 11 CINTESIS@RISE, Alameda Professor Hernâni Monteiro, 4200-319 Porto, Portugal

**Keywords:** paediatric thyroid nodules, the Bethesda system for reporting thyroid cytology, thyroid cytology, TBSRTC, paediatric cytology, FNAC, Bethesda system

## Abstract

Background: To evaluate the performance of TBSRTC through multi-institutional experience in the paediatric population and questioning the management recommendation of ATA Guidelines Task Force on Paediatric Thyroid Cancer; Methods: A retrospective search was conducted in 4 institutions to identify consecutive thyroid FNAC cases in paediatric population between 2000 and 2018. Following the 2nd TBSRTC, the risk of malignancy ratios (ROMs) was given in ranges and calculated by 2 different ways. Sensitivity, specificity, PPV, NPV and DA ratios were calculated using histologic diagnosis as the gold standard; Results: Among a total of 405 specimens, the distribution of cases for each category was, 44 (11%) for ND, 204 (50%) for B category, 40 (10%) for AUS/FLUS, 36 (9%) for FN/SFN, 24 (6%) for SFM and 57 (14%) for M categories. 153 cases have a histological diagnosis. The ratio of surgery was 23% in ND, 16% in the B, 45% for AUS/FLUS, 75% for SFN/FN and 92% for SFM and 75% in M categories; Conclusions: The data underlines the high ROM values in paediatric population which might be clinically meaningful. The high rate of malignancy of the cohort of operated patients (50%) also underlines the need of better preoperative indicators for stratification. Considering that more than half of the nodules in AUS/FLUS category were benign, direct surgery recommendation could be questionable as proposed in ATA 2015 guidelines.

## 1. Introduction

Thyroid nodules are uncommon in paediatric population and most of them are expected to be benign. The estimated prevalence and annual increase of thyroid cancer (TC) in paediatric age have been reported to be 2% and 1.1%, respectively and in a similar range as in the adult population. Differently from adult population, the literature load appoints to a wide range (10–50%) of average incidence of thyroid cancer in paediatric thyroid nodules [1,2,3,4].

Paediatric TC have a distinct molecular profile: gene rearrangements are significantly higher whereas point mutations were found to be less frequent than in adults [5]. Papillary thyroid carcinoma (PTC) is the most common TC in both paediatric and adult population, but the frequency of the most common variants of PTC are different in the paediatric population from adults. At clinical presentation paediatric differentiated TCs are prone to be more aggressive with a high frequency of local or distant metastasis at the time of the diagnosis, despite the paradox of showing more indolent course compared to adult population [3]. Due to these peculiarities, distinct profile and to the restricted data in paediatric thyroid nodules in the literature, the differential management of these nodules in comparison with the ones from adults have become at the centre of the literature. As of this, in 2015, for the first time, specific guidelines for paediatric thyroid nodules were released. [3]. In this guideline, fine needle aspiration cytology (FNAC) was endorsed as a primary diagnostic method, as in adult population. Curiously, despite all the molecular, clinic-pathological and treatment differences between the paediatric and adult patients, the Bethesda System for Reporting Thyroid Cytology (TBSRTC) [6] remained as the recommended reporting system in the cytologic evaluation of paediatric thyroid nodules. At present, there are only limited data available regarding the performance of TBSRTC on childhood onset thyroid nodules [3,6]. Thus, our group aimed to present a real-life scene merging data from 4 institutions of 2 countries analysing the efficacy and utility of TBSRTC on paediatric thyroid nodules, to be able to understand the suitability of TBSRTC for this group of patients and the management of “ATA Guidelines Task Force on Paediatric Thyroid Cancer” for the paediatric thyroid nodules.

## 2. Materials and Methods

The study cohort included thyroid FNAC cases collected from 4 institutions: Hospital de São João, Department of Pathology/Cancer Signalling and Metabolism, i3s (Porto, Portugal); Acibadem University, Department of Cytopathology, (Istanbul, Turkey); Çukurova University, Department of Pathology (Adana, Turkey); Trakya University, Department of Pathology (Edirne, Turkey). A retrospective search of the electronic medical record system was conducted at each institution to identify consecutive thyroid FNAC cases in paediatric population admitted to the pathology laboratories between 2000 and 2018. All patients were <21 years of age at presentation. All cases were categorized with TBSRTC: non-diagnostic (ND), benign (B), atypia of undetermined significance/follicular lesion of undetermined significance (AUS/FLUS), follicular neoplasm/suspicious for follicular neoplasm (FN/SFN; including cases with Hürthle cell/oncocytic features), suspicious for malignancy (SFM), and malignant. For the cases reported before 2007, diagnoses were adjusted to the corresponding TBSRTC category. The data of each cohort was collected from each institution and included the age, gender, size of the index nodule, cytological diagnosis based on TBSRTC, surgical pathology follow-up (SPFU). Only those surgical resection cases for which the resected nodule was deemed to correlate with the thyroid nodule sampled by FNAC were included in our cohort. Incidental papillary thyroid microcarcinoma cases within the surgical resection specimen were not included in the statistical analyses as well as the cases with repeat FNAC.

Surgical specimens with a histological diagnosis of malignancy, particularly those with a diagnosis of follicular variant of papillary thyroid carcinoma, were further revised to identify cases with features of non-invasive follicular thyroid neoplasm with papillary nuclear features (NIFTP) [7]. Data collection and statistical analyses were performed using Microsoft Excel software (Microsoft, Redmond, Wash) and Standard descriptive analysis was performed. Following the 2nd TBSRTC, the risk of malignancy ratios (ROMs) was given in ranges and calculated by 2 different ways. (i) First range was obtained by dividing (M + LMP) cases by the total number of FNAC and second range dividing the (M + LMP) cases by the total number of cases with SPFU, respectively, (ii) Using the same methodology but excluding NIFTP on ROM calculation (NIFTP ≠ CA), for each category. Sensitivity, specificity, positive and negative predictive values (PPV and NPV, respectively) and diagnostic accuracy (DA) ratios were calculated using histologic diagnosis as the gold standard. The ratios were determined based on three categorical approaches and named as categories I, II and III. In category I, only SFM and M cases were regarded as a positive result. FN/SFN, SFM and M cases were inserted as positive tests in category II. Lastly, AUS/FLUS, FN/SFN, SFM and M cases were considered positive test in category III.

This study was conducted with the approval of the institutional review boards (or its equivalents) of all institutions involved in this study (this study was approved by the Research Ethics Committee of the Hospital São João. No:66/19).

## 3. Results

A total of 405 thyroid FNA specimens (corresponding to 405 patients) were obtained between 2000–2018. Patient ages ranged from 3 to 21 years; the average age was 16 with a female predominance (F/M-308/97). The mean size of the nodules was 22.6 mm, 6% (11 cases) had a tumour larger than 40 mm and 13% (22 cases) had tumours smaller than 10 mm. Of the 405 cases, 153 underwent surgical intervention, and 55% of them have a total thyroidectomy.

Table 1 shows the demographic data of the multi-institutional case cohort analysis. Most cases analysed (58%) were from the Hospital de São João (Porto) (HSJ) and only 6 cases (1%) were from the University of Trakya. In all the 4 institutions, there is a prevalence of females, as well as those under 18 years of age.

153 cases have a histological diagnosis (Table 2). The ratio of surgery was 45% for AUS/FLUS, 75% for SFN/FN and 92% for SFM and only 16% in the Benign category, which is expected since most of the cases in this category have no surgical indication. In ND category 23% of the cases were subjected to surgery.

Of the patients undergoing surgery, 76 of them (49.7%) had a benign or low-ma-lignancy potential (LMP) lesion, whereas 50.3% of them had a classical malignancy (Table 3). Regarding the biological behaviour of the different diagnostic entities by histology of the 10 cases referred to ND category, 7 (70%) were benign and 3 (30%) malignant. For the 33 cases referred to B category, 28 (85%) were confirmed as benign, 5 cases (15%) were malignant. Of the 18 operated cases diagnosed as AUS/FLUS category, 12 (67%) were benign, 4 (22%) malignant and 2 (11%) diagnosed as LMP lesions (borderline). FN/SFN showed a distribution between benign and malignant of 13 and 12 respectively, the remaining 2 cases (7%) were diagnosed as LMP. Of the 22 cases diagnosed as SFM, 16 were malignant (73%), 3 (14%) were of LMP and 3 (14%) were benign. Finally, M presented 37 (86%) malignant cases, whereas 5 cases (12%) were come out as benign and 1 (2%) as low malignancy potential (Table 3).

ROM was evaluated in two types of range-based calculation. First range is between underestimated (M + LMP/total FNAC cases by category) and overestimated (M + LMP/total SPFU cases by category) ROMs, whereas the second reflects the same range but excluding NIFTP (7 cases were reclassified as NIFTP after histologic revision). In both ways, ND and B categories were found to have a higher ROM than the suggested ranges of TBSRTC. (Table 3).

In Table 4, brief literature review of the distribution of TBSRTC categories based on ROMs was summarized to be able to compare the current study results, as well as the TBSRTC. Although there are slightly differences in the calculation of ROM between the different studies, most of them do not take in account the diagnosis of NIFTP and use the number of positive patients divided by all biopsied patients to estimate the ROM.

**Figure 1:**
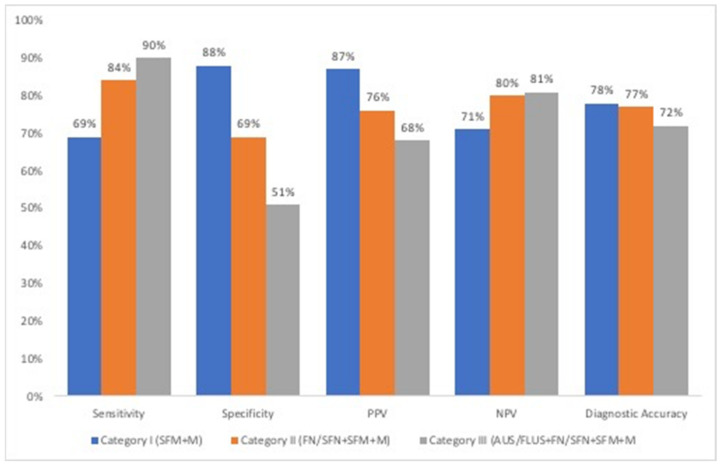
Comparisons derived by different thresholds of FNAB interpretations.

Since the AUS/FLUS and FN/SFN are the most accepted grey-zone categories of TBSRTC, sensitivity, specificity, PPV, NPV and diagnostic accuracy values were evaluated in categorical fashion (categories “I”, “II” and “III”) to be able to evaluate the idea of compressing the grey zone categories in one category, and to clarify the general diagnostic performance of TBSRTC (Figure 1).

To have a greater specification of histopathology, we compared the various TBSRTC categories with the various types of histopathological diagnosis (Table 5). In SFM and M categories 12 false positive cases were found, 6 of them belong to M categories, of those 3 cases were FNH, 2 cases were CLT and 1 case was NIFTP. Of 6 false positive cases in SFM category, 3 cases were FNH, and 3 cases were NIFTP. The 5 false negatives in B category were turned out to be PTC. The ratio of NIFTP cases were highest in SFM (14%; 3/22); 1 case of WT-UMP was assigned to the SFN/FN category.

## 4. Discussion

As in the adult population, thyroid cancer is the most common endocrine malignancy in childhood [5]. For many years, paediatric thyroid nodules were being treated based on the guidelines for adult thyroid nodules. Due to the multi-layered unique profile of paediatric thyroid cancer, the first ATA guidelines were released targeting the paediatric population with recommendation to use TBSRTC as first step in the evaluation of paediatric thyroid nodules [3,6]. Although the reporting system remained the same as recommended for adult population, the ATA 2015 guidelines promoted different management recommendations specifically for the undetermined categories of TBSRTC. Thus, our group decided to investigate the implied recommendations of ATA through the detailed analyse of TBSRTC performance in a large cohort of paediatric thyroid nodules [3,6].

In the current study we found that 20% (24 + 57) of 405 FNAC cases were assigned in SFM and M categories, whereas 50% (77/153) of the nodules who had SPFU were ultimately diagnosed as cancer. Our data is different from previous studies that showed lower rates of malignancy in paediatric population [15,16,17]. Based in a literature search for TBSRTC studies in paediatric patients, the ROMs presented a high variability by institution and categories [4,8,9,10,11,12,13,14]. This can be attributed to the fact that there are limited number of studies for paediatric patients and the studies were done based in a restricted number of patients. It is also noted by the TBSRTC 2nd edition that when using total number of SPFU as denominator ends with overestimation, whereas using total FNAC numbers as denominator ends with underestimation [6]. To refrain this extrapolation, our group analysed the cohort in both ways, since the real ROM is expected to be in the midrange of two edges. Even by the midrange approach, our ROMs were found higher than the suggested ROMs by TBSRTC except for M category. The brief literature documentation also supports, in paediatric thyroid nodules, higher ROMs in all categories in comparison with TBSRTC (Table 4) [4,8,9,10,11,12]. Although the majority of the previous studies were based in cohort with less than 100 cases of SPFU, a wide range of ROM was evident between our cohort and that reported in the literature review (Table 3 and Table 4). The second midrange analysis was done by with or without NIFTP, and, even in this case, all the ROMs except the M category were found higher than the ROMs of TBSRTC with and without NIFTP [7]. M category was diluted by 2 cases of CLT, 3 cases of FNH and 1 case of NIFTP which were false positives, assigned as M in our cohort. CLT is a well-known source of false positive cases in thyroid cytology, whereas FNH can be challenging whenever thyroid goes to stromal degeneration. NIFTP cases can be accepted as false positive given the fact that recommendation of surgery is lobectomy. It should be noted that the last two entities also were the cause of false positive in SFM cases. On the other hand, the most common false negative cause was PTC, as expected. The five PTCs assigned as benign correspond to two cases of classical variant with a highly cystic nature, two cases of FV-PTC and 1 case of miPTC. In addition, a case of Hodgkin lymphoma was reported as ND.

It is noteworthy to mention that in our work, besides the large number of cases included, it also reflects a real-life scene involving four institutes from two countries with the inherent heterogeneity of diagnostic thresholds for assessing the FNAC and SPFU cases. The higher ROMs found in the current study, even in midrange approach, might support the idea of a greater risk of malignancy in the paediatric population with thyroid nodules, and surgery may be favoured for nodules reported in the grey zone categories as recommended by ATA Guidelines Task Force on Paediatric Thyroid Cancer [3].

Sensitivity, specificity, PPV, NPV and DA were comparable with the review of the literature, particularly when considering our categorical approach [11,12,13,14]. Sensitivity and NPV are most increased in category “III”, showing the efficacy of TBSRTC as a screening test. On the other hand, in the same category, PPV and specificity shows a significant decrease (67% and 51%) that indicates the loss of diagnostic power of the reporting system. These 3 categorical approaches also did not support the idea of a “compressed 4-category of TBSRTC” that has been suggested by some authors for paediatric population [11,18]. Category “II” reflects the results when AUS/FLUS merged with benign cytologic diagnosis, whereas “III” reflects the scenario when AUS/FLUS merged with FN/SFN. Given the fact that more than half of the AUS/FLUS cases were benign, if the AUS/FLUS category would merge with FN/SFN, it would dilute the ROMs in FN/SFN category. Yet, although not performed in the current cohort, the use of molecular testing has been suggested to have value in the management of these indeterminate thyroid aspirates in paediatric patients [8].

Our series with a large cohort and high surgery rates in ND, B and undetermined categories, provides more clear results about the use of TBSRTC in paediatric population and contrasts with the previous reports in the literature [4,8,9,10,11,12]. The data underlines the high ROM values in paediatric population which might be clinically meaningful. The high rate of malignancy also underlines the need of better preoperative indicators for stratification.

Of course, being the 4 institutes tertiary centres, it might be the reason of a possible bias for the high-risk malignant nodules, besides the lack of routine evaluation of on-site adequacy in majority of the case cohort might also have some contribution, especially in ND category. Considering that more than half of the nodules in AUS/FLUS category were benign, direct surgery recommendation could be questionable as proposed in ATA 2015 guidelines [3]. The use of ultrasound-based risk stratification systems to manage paediatric patients was previous validated [15] but until this moment the results are quite controversial with some studies showing a suboptimal performance for indication for FNAC and consequent cancer detection [19,20].

To the best of our knowledge, the current study presents one of the largest multi-institutional case cohort study of paediatric nodular thyroid disease and may provide a significant contribution to tailor the future recommendations for a better preoperative risk assessment in conjunction with cytopathology reporting and reduce the over treatment in paediatric nodular thyroid disease.

## Figures and Tables

**Table 1:** Demographic data of the cases from each institute.

Institution	FNAC ^1^		SPFU ^2^		Gender		Age (years)	
	n	%	n	%	F	M	<18	18–21
HSJ	236	58	78	52	186	50	152	84
Acibadem Univ.	136	34	43	28	98	38	70	66
Çukurova Univ.	27	7	26	17	19	8	27	0
Trakya Univ.	6	1	6	4	5	1	6	0
Total	405	100	153	100	308 (76%)	97 (24%)	255 (63%)	150 (37%)

^1^ FNAC: Fine-Needle Aspiration Cytology, ^2^ SPFU: Surgical Pathology Follow-Up.

**Table 2:** The Demographic data of the cases subject to SPFU according to TBSRTC categories.

Categories of TBSRTC	SPFU/FNAC (%)	Gender		Number of Cases by Age Range		Nodule Size, mm (Median-IQR)
		F	M	<18	18–21	
ND ^1^	10/44 (23)	7	3	8	2	27.5 (13.7–39)
B ^2^	33/204 (16)	32	1	19	14	29 (12–31)
AUS/FLUS ^3^	18/40 (45)	13	5	12	6	19 (10–25)
FN/SFN ^4^	27/36 (75)	17	10	21	6	20.5 (13.5–35)
SFM ^5^	22/24 (92)	16	6	15	7	12.5 (9.5–22.5)
M ^6^	43/57 (75)	29	14	28	15	20 (13–32)
Total	153/405 (38)	114	39	103	50	-

^1^ ND: Non-Diagnostic, ^2^ B: Benign, ^3^ AUS/FLUS: atypia of undetermined significance/follicular lesion of undetermined significance,^4^ FN/SFN: follicular neoplasm/suspicious for follicular neoplasm,^5^ SFM: Suspicious of Malignancy, ^6^ M: Malignant.

**Table 3:** Distribution of TBSRTC categories based on SPFU, biological behavior and ROMs.

	ND	B	AUS/FLUS	FN/SFN	SFM	M
Total FNACs (n = 405), Nº (%)	44 (11)	204 (50)	40 (10)	36 (9)	24 (6)	57 (14)
Surgical pathology follow-up (SPFU) (n = 153), Nº/each category (%)	10/44 (23)	33/204 (16)	18/40 (45)	27/36 (75)	22/24 (92)	43/57 (75)
Benign SPFU (n = 68), Nº	7	28	12	13	3	5
Low-Malignancy Potental SPFU (n = 8), Nº	0	0	2	2	3	1
Malignant SPFU (n = 77)	3	5	4	12	16	37
ROM [(M + LMP)/FNAC − (M + LMP)/SPFU)], %	7–30	2.5–15	15–33	39–52	79–86	67–88
ROM suggested by TBSRTC	5–10	0–3	10–30	25–40	50–75	97–99
ROM (M/FNAC − M/SPFU), % NIFTP ≠ CA	7–30	2.5–15	10–22	33–44	66–73	65–86
ROM suggested by TBSRTC NIFTP ≠ CA	5–10	0–3	6–18	10–40	45–60	94–96

**Table 4:** Brief literature review of the distribution of TBSRTC categories based on ROMs [4,8,9,10,11,12].

Reference	Country	Year	N (Total FNA)	ND	B	AUS/FLUS	FN/SFN	SFM	M
				ROM					
Monaco et al. [8]	USA	2012	96	0%	7%	28%	58%	100%	100%
Gupta et al. [9]	USA	2013	64	8%	14%	40%	100%	40%	100%
Norlen at al. [4]	Australia	2015	35	0%	0%	22%	100%	100%	100%
Lale et al. [10]	USA	2015	78	10%	0%	50%	47%	100%	100%
Amirazodi et al. [11]	Canada	2015	65	0%	16%	67%		71%	100%
Cherella et al. [12]	USA	2019	430	11%	0.7%	44%	71%	73%	97%
Jiang et al. [13]	USA	2021	203	13.8%	4.7%	22.7%	35.7%	83.3%	100%
Jia et al. [14]	USA	2021	575	0.0%	0.8%	15.6%	54.5%	100%	100%
**TBSRTC**	**USA**	**2017**	**-**	**5–10%**	**0–3%**	**6–18%**	**10–40%**	**45–60%**	**94–96%**
Current study	**Portugal-Turkey**	**2021**	**405**	**7–30%**	**2.5–15%**	**15–33%**	**39–52%**	**79–86%**	**67–88%**

**Table 5:** Distribution of SPFU cases based on the histological types and TBSRTC categories.

Categories of TBSRTC	Benign (n = 68)					Low-Malignity Potential (n = 8)		Malignant (n = 77)				
	CLT	FNH	DHG	FA	OA	NIFTP	WDT-UMP	WDC-NOS	PTC	MTC	Lymphoma (Hodgkin)	Total
ND	2	5	0	0	0	0	0	0	2	0	1	10
B	3	16	1	8	0	0	0	0	5	0	0	33
AUS/FLUS	2	4	0	6	0	2	0	0	4	0	0	18
FN/SFN	0	5	1	6	1	1	1	1	11	0	0	27
SFM	0	3	0	0	0	3	0	0	15	1	0	22
M	2	3	0	0	0	1	0	0	37	0	0	43
Total	9	36	2	20	1	7	1	1	74	1	1	153

CLT: chronic lymphocytic thyroiditis, FNH: Follicular nodular hyperplasia, DHG: dishormonogenetic goiter, FA: follicular adenoma, OA: oncocytic adenoma, NIFTP: non-invasive follicular thyroid neoplasm with papillary nuclear features WDT-UMP: well differentiated tumour with uncertain malignant potential, WDC-NOS: Well-differentiated carcinoma-not otherwise specified.
